# Evaluating the efficiency of a novel rotary reactor for the treatment of acid mine drainage using armored limestone

**DOI:** 10.1007/s11356-026-38360-0

**Published:** 2026-10-29

**Authors:** Nicolas Rojas-Arias, Sebastian Felipe Rojas-Arias, Cesar René Blanco-Zuñiga

**Affiliations:** 1 Dipartimento di Scienza Applicata e Tecnologia, Politecnico Di Torino, Viale T. Michel 5, 15121 Alessandria, Italy; 2 C.I. Hábitat a Vibe Company S.A.S. 110931, Bogotá, Colombia; 3 Universidad Pedagógica y Tecnológica de Colombia, Avenida Central del Norte 39-115, PO Box 150001, Boyacá, Colombia; 4 School of Mechanical Engineering, State University of Campinas (UNICAMP), Campinas, SP 13083–860 Brazil

**Keywords:** Acid mine drainage, Limestone, Rotary reactor, Water quality, Surface abrasion

## Abstract

This study evaluates the efficiency of a novel rotary mechanical system designed to treat acid mine drainage (AMD) generated by coal mining activities, aiming to reactivate limestone affected by surface armoring during neutralization process. Characterization via X-ray diffraction and scanning electron microscopy revealed the formation of goethite, siderite, and gypsum within the passivating layer developed on the limestone. The results demonstrate the effectiveness of the rotary system in mechanically removing this passivating layer, thereby restoring limestone reactivity, and significantly improving the physicochemical quality of the treated AMD. Implementing this mechanical approach enhances overall process efficiency while reducing limestone replacement and maintenance requirements.

Coal remains one of the most critical and affordable energy sources worldwide (Luo et al. 2024). In countries such as Colombia, coal mining is a major pillar of economic development and a key export sector (Carmona and Dąbkowska 2024; Jakobsen 2024). Nevertheless, its exploitation generates hazardous byproducts such as acid mine drainage (AMD), which is characterized by extreme acidity and high concentrations of iron and heavy metals, posing serious risks to surrounding soil and water resources. Over the years various methods have been developed to treat AMD, including limestone treatment; however, these methods still present various financial and sustainability implications (Wang et al. 2024). Limestone passive AMD treatments can be efficient in the long term; nevertheless, the coating formed on limestone surface can only be removed by agitation of the medium (Skousen et al. 2017). AMD forms when sulfur- and iron-rich minerals, particularly pyrite (FeS_2_), oxidize and heavy metals dissolve in tailings water (Blanco-Zuñiga et al. 2022).

Active treatment systems are widely used to remediate AMD, yet the reaction between limestone and AMD produces a passive surface layer (“armoring”) that limits neutralization efficiency, requiring frequent maintenance steps and limestone replacement (Daraz et al. 2023). Enhancing limestone reactivity in these systems would represent a significant advance in restoring water bodies impacted by coal mining. Considering the above, this study proposes a novel rotary reactor device designed to enhance the limestone reactivity used during the treatment of acid mine drainage (AMD) from coal mining activities in Boyacá, Colombia.

Dissolved oxygen (DO, ASTM D888-09), pH (SM 4500-H+B), and electrical conductivity (SM 2510B) were determined. Likewise, total Fe and sulfate concentrations were measured via spectrophotometry technique (Ferrover®, M-8008; Sulfaver®, M-8051), while alkalinity and total acidity were determined by titration with H_2_SO_4_ (0.02 N) and NaOH (0.02 N), respectively, using bromocresol green as indicator (SM-2320). The results are summarized in Table 1. The high acidity of the AMD is mainly attributed to the oxidation of sulfide minerals such as pyrite, which is common in these mineral deposits (Blanco-Zuñiga et al. 2022). According to Resolution 0631 of 2015 issued by Colombia’s Environmental Ministry, values exceed the maximum permissible limits for discharge into water bodies and sewers, thereby requiring treatment.

**Table 1:** Physicochemical characteristics of the AMD samples used in this study and reference values for water quality according the Resolution 0631/2015

Physicochemical parameter		0631/2015 Legislation	AMD	Treated AMD with limestone			
				Unarmored		Armored	
				10 mm	5 mm	10 mm	5 mm
pH	[-]	6.0–9.0	2.72	7.02−7.11	7.13–7.21	7.03–7.13	7.01–7.15
DO	[mg · L^−1^]	-	7.24	7.14–7.45	6.63–7.10	7.09–7.40	7.04–7.07
Conductivity	[mS · cm^−1^]	-	2.49	3.15–4.35	3.37–3.58	2.81–3.83	2.76–3.51
Acidity	[mg · L^−1^]	Report	1761.3	10.0–12.3	6.8–13.9	11.6–14.2	7.8–9.8
Alkalinity	[mg · L^−1^]	Report	0	60–63	59–63	52–63	55–72
Fe _Total_	[mg · L^−1^]	2.0	295	0.03–0.17	0.04–0.06	0.04–0.09	0.06–0.07
SO_4_^−2^	[mg · L^−1^]	1200	2500	2000–2350	1250−1500	2050−2250	875−1750

Limestone samples collected from the same source were crushed and sieved into two particle size fractions. Grain-size analysis yielded median particle diameters (D_50_) of 10 and 5 mm for the coarse and fine fractions, respectively. To investigate the armoring effects, representative samples from each size fraction were immersed in acid mine drainage (AMD) for 4 weeks. The mineralogical composition of the limestone surface prior to and following armoring was examined using X-ray diffraction (XRD), and the corresponding diffraction patterns are presented in Fig. 1. Rietveld refinement of the unarmored limestone revealed a mineralogical composition of 80.42% CaCO_3_, 5.83% MgCO_3_, and 13.75% SiO_2_. In contrast, the analysis of the armored surface confirmed the formation of CaSO_4_ · n(H_2_O), FeO(OH), and FeCO_3_, which is consistent with the secondary mineral precipitates commonly reported in AMD environments (Mosai et al. 2024).

**Fig. 1:**
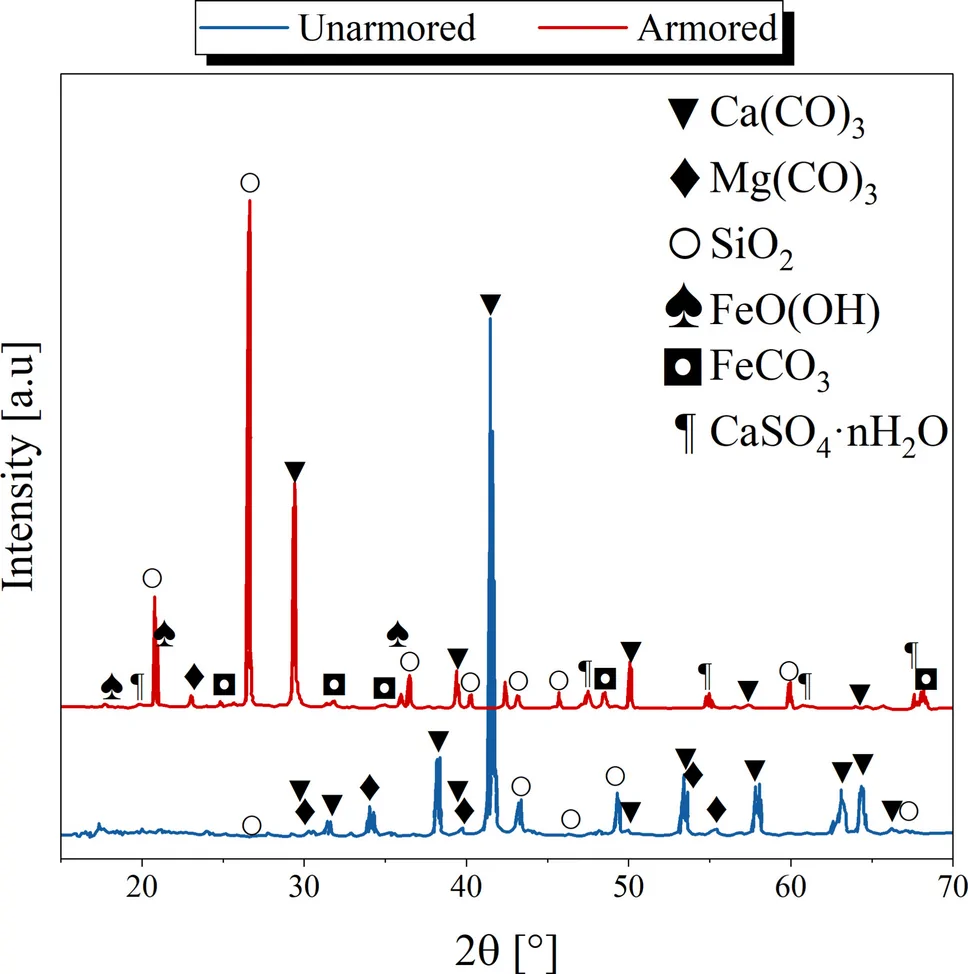
XRD patterns for unarmored and armored limestone samples used in this study

The AMD samples were treated in a novel rotary reactor consisting of several components, as illustrated in Fig. 2. The cylindrical reactor, including its front and rear end caps, was perforated with randomly distributed 2.0-mm-diameter openings across its entire surface. This configuration allowed AMD to enter the reactor by immersion while facilitating the removal of precipitated solids and other materials released during limestone armoring. The reactor featured an internal diameter of 15.24 cm and a length of 16.4 cm, corresponding to an internal working volume of approximately 3.0 L. It was loaded with 0.75 kg of limestone, filling approximately 45 vol. % of the reactor. The remaining 55 vol. % was left as free space to allow air ingress and the release of CO_2_ generated during the neutralization reaction. Furthermore, external paddles kept the resulting precipitates in suspension and promoted thorough mixing of the acidic medium, thereby enhancing contact between the AMD and limestone. Although the AMD container had a total capacity of 5.0 L, only 3.0 L were used in each experiment to minimize splashing losses during agitation and reactor rotation. Treatment was sustained until the AMD reached a pH of approximately 7.0.

**Fig. 2:**
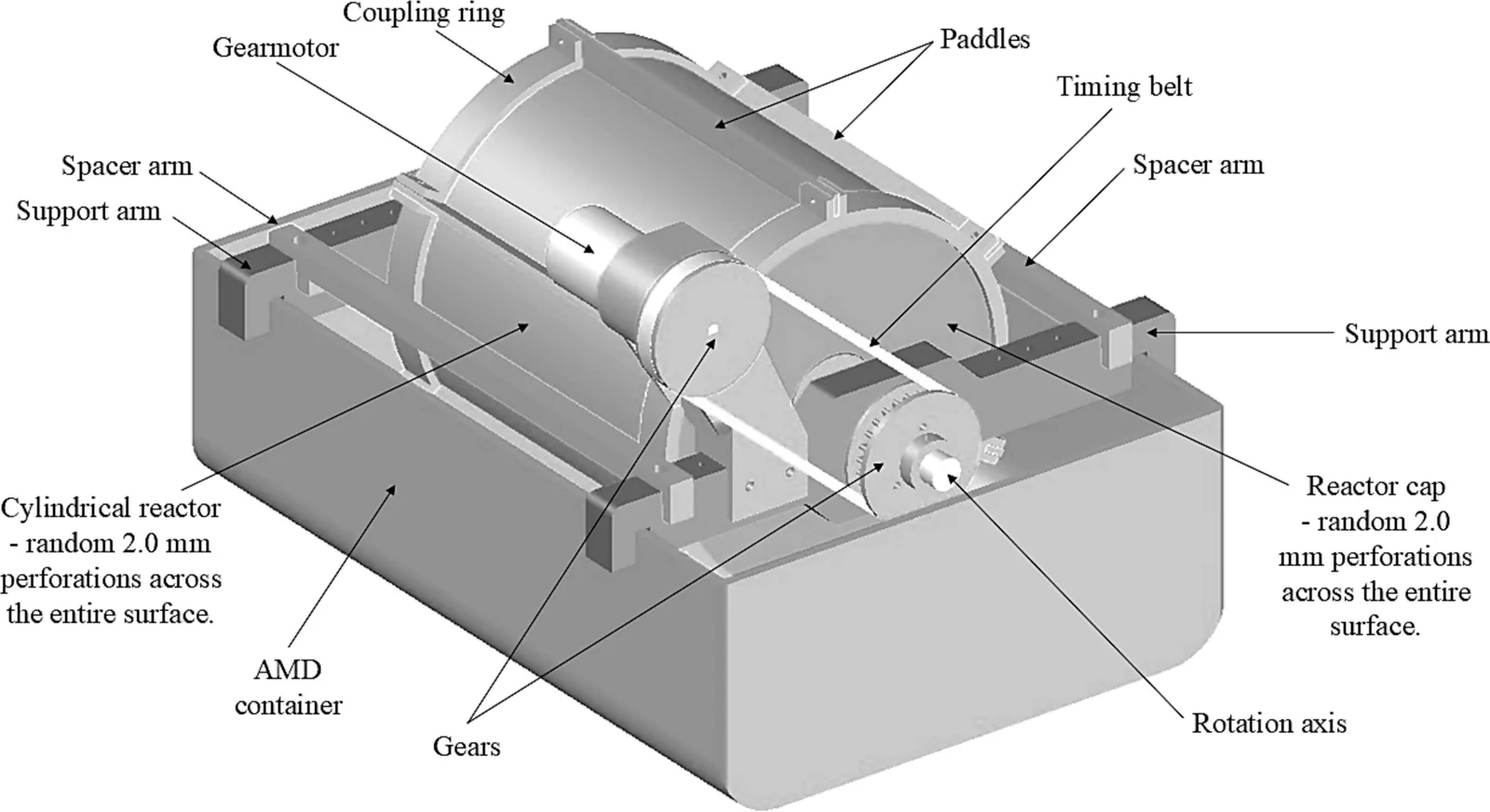
Schematic diagram of the rotary reactor used in this study. The reactor had an internal diameter of 15.24 cm and a length of 16.4 cm, corresponding to an internal working volume of approximately 3.0 L

All experiments were conducted using a constant AMD-to-limestone mass ratio of 4:1, in a controlled environment compliant with ISO 14644. The average room temperature was maintained at 19.4 ± 1.0 °C, under an atmospheric pressure of 740 hPa at an altitude of 2800 m above sea level (m.a.s.l.).

A total of 12 experimental conditions were evaluated by cross-examining three rotation speeds (20, 30, and 40 rpm), two particle size fractions, and both unarmored and pre-armored limestone samples. Three independent experiments were performed for each condition. The pH was monitored at 15 min intervals using a SCHOTT Handylab pH-11 pH meter. The resulting pH evolution curves are presented in Fig. 3. The time required to reach a pH of 7.0 under the different conditions investigated in this study is summarized in Table 2.

**Fig. 3:**
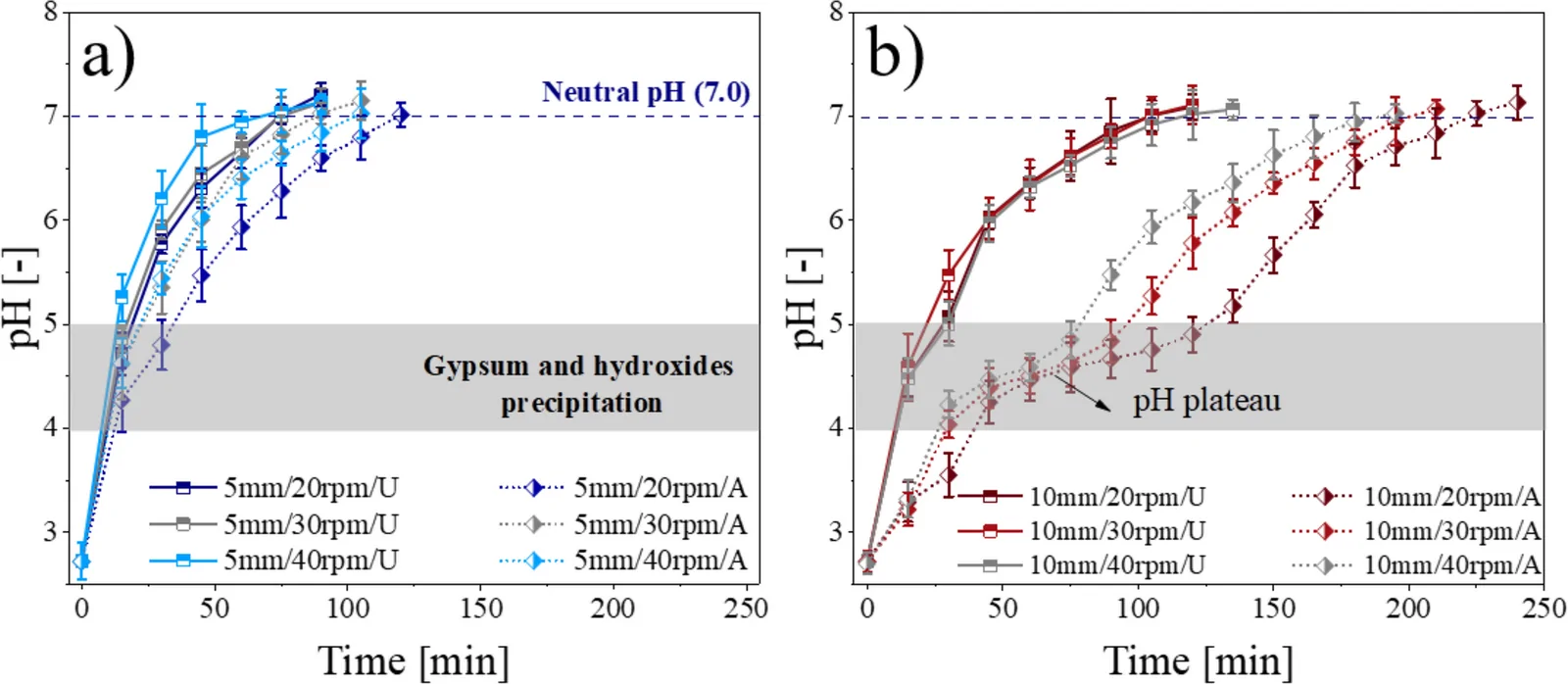
pH evolution during AMD treatment using: **a** fine and **b** coarse limestone samples under unarmored (U) and armored (A) conditions

**Table 2:** Effect of the different conditions investigated in this study on the time required to reach a pH of 7.0 and the removal of total Fe and dissolved sulfate (SO_4_^−2^) from AMD

Condition	Time required	Fe_Total_ removal	SO_4_^−2^ removal
	[min]	[%]	[%]
5 mm/20 rpm/U	73 ± 4	99.98 ± 0.01	40.0 ± 4.9
5 mm/30 rpm/U	75 ± 10	99.98 ± 0.01	49.7 ± 1.9
5 mm/40 rpm/U	65 ± 7	99.99 ± 0.00	50.0 ± 6.8
5 mm/20 rpm/A	120 ± 6	99.97 ± 0.01	30.0 ± 3.2
5 mm/30 rpm/A	90 ± 12	99.97 ± 0.00	43.2 ± 2.0
5 mm/40 rpm/A	105 ± 16	99.97 ± 0.02	65.0 ± 3.2
10 mm/20 rpm/U	104 ± 18	99.94 ± 0.01	6.0 ± 5.9
10 mm/30 rpm/U	104 ± 9	99.95 ± 0.03	15.5 ± 7.8
10 mm/40 rpm/U	110 ± 15	99.98 ± 0.01	20.0 ± 3.9
10 mm/20 rpm/A	222 ± 13	99.96 ± 0.00	10.1 ± 6.0
10 mm/30 rpm/A	195 ± 10	99.97 ± 0.01	11.8 ± 2.4
10 mm/40 rpm/A	185 ± 15	99.99 ± 0.00	18.0 ± 3.5

For the unarmored limestone, rotation speed had little influence on neutralization kinetics. AMD treatment with coarse particles required 104–110 min to reach pH 7.0, whereas finer fractions achieved the same value within 65–75 min. This behavior is attributed to the higher specific surface area of the finer particles, which provides more reactive sites and accelerates the neutralization reaction.

The use of armored limestone significantly extended treatment times. Coarse armored particles exhibited the lowest efficiency requiring 185–222 min to reach pH 7.0, whereas the finer particles achieved neutrality within 90–120 min. In both cases, elevated rotation speeds shortened treatment durations by mechanically removing the passivation layer, thereby exposing fresh reactive surfaces and maintaining limestone reactivity.

The surface morphology of the limestone was examined via scanning electron microscopy (SEM). The secondary-electron images presented in Fig. 4 revealed the presence of dense precipitates with plate and columnar morphologies on the armored particles. Energy-dispersive X-ray spectroscopy (SEM-EDX) (Fig. 4c) and XRD (Fig. 1) analyses of the armored layer confirmed the formation of CaSO_4_ · n(H_2_O), FeO(OH), and FeCO_3_. SEM-EDX analysis of the limestone particles after AMD treatment revealed reduced Fe concentrations and the absence of S on the surface. These findings indicate that the secondary precipitates formed during armoring were effectively removed thereby exposing a fresh reactive limestone surface.

**Fig. 4:**
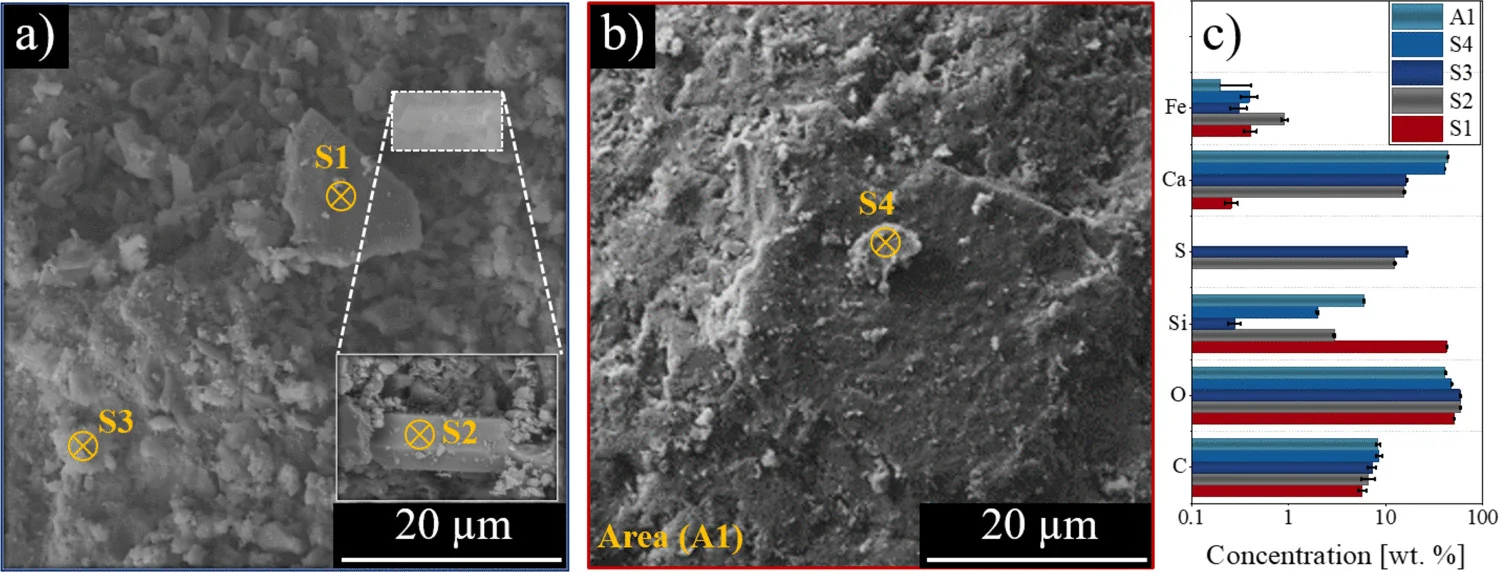
Surface morphology analysis by secondary-electron SEM imaging for the armored limestone **a** before and **b** after AMD treatment in rotary system. In **c**, elemental composition by SEM-EDX of the precipitates is highlighted in Fig. 3**a** and **b**

The AMD treated with coarse particles exhibited a transient plateau at pH ~ 4.5 between 45 and 90 min. This behavior is attributed to the precipitation of gypsum and iron hydroxides, which typically occurs within the pH range 4–5 (Cheong et al. 2022), which temporarily passivates the limestone surface. In this study, the most efficient experimental condition—AMD treated with particles of 5 mm and 40 rpm rotation speed—resulted in a 99.56 ± 0.21% reduction in acidity and 99.97 ± 0.02% removal of total Fe. Despite a slight decrease in dissolved oxygen (DO) concentration (from 7.24 to 7.04 ± 0.03 mg · L^−1^), the measured values remained close to the DO saturation level reported for the zone studied, i.e., 7.5 mg · L^−1^ (Rojas Arias et al. 2021). Furthermore, the sludge generated during AMD treatment was recovered via filtration and dried at 105 °C for 24 h. The process produced approximately 8.9 ± 0.5 g · L^−1^ of sludge, primarily due to the continuous precipitation of secondary minerals and the abrasion-driven removal of armoring deposits from the limestone surface.

In summary, coal mine drainage from Boyacá, Colombia, was effectively treated using a rotary reactor designed to sustain limestone reactivity. Although surface armoring initially decreased neutralization efficiency; nevertheless, mechanical rotation effectively removed the passivation layer through abrasion, continuously exposing fresh reactive surfaces. This approach significantly enhances treatment performance while reducing both maintenance frequency and limestone replacement rates in active AMD remediation systems.

## Data Availability

Data shall be made available on request.
